# Influence of Maturity Stage at Harvest on the Fruit Quality and Volatile Organic Compounds of “Legacy” Blueberry

**DOI:** 10.1002/fsn3.71792

**Published:** 2026-04-16

**Authors:** Wenkuan Zhang, Zhihua Wang, Wenhui Wang, Chaoshuang Jia, Yang Wang, Shumin Zhang, Qiang Yue, Yanmin Du

**Affiliations:** ^1^ Institute of Pomology, Chinese Academy of Agricultural Sciences Xingcheng China; ^2^ Key Laboratory of Germplasm Resources Utilization of Horticultural Crops Ministry of Agriculture and Rural Affairs Xingcheng China; ^3^ Key Laboratory of Fruits Storage and Processing of Liaoning Province Xingcheng China

**Keywords:** “legacy” blueberry, multivariate statistics, nutritional quality, volatile organic compounds

## Abstract

This study systematically investigated the physicochemical properties and aroma dynamics of highbush blueberry ( *Vaccinium corymbosum* “Legacy”) across five maturity stages (I: green to V: dark blue) using headspace solid‐phase microextraction coupled with gas chromatography–mass spectrometry (HS‐SPME‐GC–MS). The fruit diameter, fresh fruit weight, soluble solids content, soluble sugars, and vitamin C content of these blueberries were found to increase significantly with maturation, whereas the firmness and titratable acidity decreased. Seventy‐seven volatile organic compounds (VOCs), predominantly consisting of aldehydes (59.09%–85.18%) and alcohols, were identified. The diversity of VOCs decreased from 65 in Maturity Stage I to 38 in Maturity Stage V, although aldehydes such as (*E*)‐2‐hexenal (which peaked at 1292.81 μg/kg in Maturity Stage I) remained consistently present across the maturation stages. Orthogonal partial least squares discrimination analysis identified 10 differential volatile metabolites, including (*E*)‐2‐hexenal and hexanal, that distinguished between maturity stages. Odor activity values revealed 17 key aroma contributors, notably hexanal (floral), *β*‐myrcene (peppery), and (*E*)‐2‐hexenal (green). Fruits at Maturity Stage IV exhibited the most intense aroma and optimal quality, characterized by their light blue peel, high soluble solids content (14.66%), balanced acidity (0.615%), and rich fruity notes. These findings establish distinct volatile signatures for each maturity stage and serve as objective biochemical markers to optimize harvest timing in blueberry cultivation. Furthermore, these stage‐specific profiles provide a scientific basis for raw material grading in product processing, guiding the targeted selection of fruits for fresh market distribution or specific processed products such as juices and fermented wines.

## Graphical abstract

This study systematically maps the dynamic changes in physicochemical properties and volatile organic compounds (VOCs) of highbush blueberries across five maturity stages. By identifying key aroma contributors and specific biochemical markers, these findings provide a solid scientific basis for optimizing harvest timing, fresh market distribution, and fruit processing.

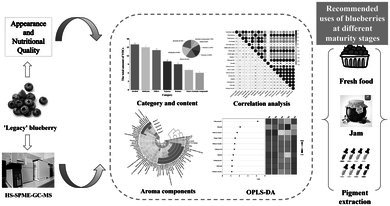

## 1 Introduction

Blueberry (*Vaccinium* spp.), a perennial shrub in the Ericaceae family, is widely known as the “King of Berries” and is occasionally referred to as bilberry in certain regions. Originally native to North America, blueberries are now globally cultivated, with large production centers in the United States and Chile catering to demand in Europe, Asia, and other regions (Gong et al. 2025). “Legacy” blueberries, a late‐maturing variety of northern highbush blueberry, were created through hybridization by the United States Department of Agriculture in 1993. Recently, China has witnessed a substantial increase in blueberry cultivation, with extensive plantations established in northeast China, Shandong, Guizhou, and other areas (Li, Liu, et al. 2025). Blueberry fruit is valued for its sweet–sour flavor, rich aroma, and beneficial nutrients, including anthocyanins, vitamins, proteins, and potent antioxidants (Chen et al. 2024). In addition to being consumed fresh, blueberries are processed into various products such as baked goods, beverages, and jams (Jiang et al. 2015; Contessa et al. 2023). Moreover, blueberry extracts, particularly anthocyanins, are widely used in health supplements and cosmetics.

Fruit aroma is a reliable indicator of flavor quality throughout the developmental stages. Blueberries are characterized by a distinctive aroma derived primarily from a complex mixture of volatile organic compounds (VOCs), such as aldehydes, alcohols, terpenes, and esters, produced during ripening. The nutritional composition and VOC profiles vary considerably with maturity (Kong et al. 2024). For instance, recent studies on other berry crops, such as red currants, have clearly demonstrated that the accumulation of specific biochemicals, particularly phenolic compounds and organic acids, is highly dependent on the ripening stage (Berk et al. 2020). Headspace solid‐phase microextraction coupled with gas chromatography–mass spectrometry (HS‐SPME‐GC–MS) is the preferred method for analyzing fruit VOCs. It offers high sensitivity, requires minimal sample preparation, and operates without solvents, making it particularly suitable for complex matrices such as fruit aromas. Researchers have widely used this method to identify aroma components in various fruits, such as peach, plum, and orange (Zheng et al. 2023; Wang, Wang, et al. 2023; Sun et al. 2024; Yuan et al. 2024; Chen et al. 2025; Martínez et al. 2022).

Several researchers have applied this method to explore blueberry aromas. Qiao et al. (2024) analyzed seven cultivars of Rabbiteye blueberries from Guizhou using HS‐SPME‐GC–MS and identified 46 aroma compounds classified into seven categories, with aldehydes being the most abundant group across all varieties, followed by benzene derivatives. Similarly, Cheng et al. (2020) identified and quantified 28 VOCs, including five esters, 11 terpenes, three aldehydes, six alcohols, and three volatile phenols, in eight blueberry cultivars from central China. These studies underscore the distinct VOC profiles among different blueberry varieties. Furthermore, Qian et al. (2022) compared the VOC composition of major *Vaccinium corymbosum* (northern highbush blueberry) cultivars and observed strong positive correlations among most terpenoids, except eucalyptol. Based on odor activity value (OAV) analysis, the authors proposed that branched‐chain esters, terpenes, and aldehydes are probably the primary aroma contributors across all 11 studied varieties. However, most existing studies have been limited to comparisons between two or three broad maturity categories (e.g., unripe vs. ripe) or have focused on postharvest changes. Because VOC accumulation is highly cultivar‐dependent, findings from other varieties cannot simply be extrapolated. Despite “Legacy” being a commercially dominant cultivar with high market demand, high‐resolution data tracking its continuous physicochemical and aroma dynamic changes across precisely defined maturity stages remain scarce.

The primary objective of this study was to systematically characterize the dynamic changes in the physicochemical properties and VOC profiles of “Legacy” blueberries across five precisely defined maturity stages. Specifically, we systematically compared the biochemical quality and VOCs of Stages I to III and V against Stage IV (the optimal commercial maturity benchmark). By delineating the variations in visual traits, nutritional composition, and high‐resolution aromatic fingerprints throughout maturation, this research fills a critical knowledge gap regarding the cultivar‐specific physiology of “Legacy”. Ultimately, these findings provide a vital scientific basis for determining optimal harvest windows and offer practical guidance for enhancing fruit flavor quality and optimizing postharvest handling and processing in the regional blueberry industry.

## 2 Materials and Methods

### 2.1 Plant Materials

The study focused on the northern highbush blueberry ( *Vaccinium corymbosum* “*Legacy*”) sourced from the Xingcheng Blueberry Experimental Base in Huludao city, Liaoning province. “Legacy” was selected due to its local prevalence and high commercial value, defining its ripening dynamics is essential for optimizing regional harvest management. At the experimental site, the average daytime temperature ranges from 21°C to 28°C, with a relative humidity of 70%–80% and an annual precipitation of approximately 600 mm. A standard drip irrigation system was employed to maintain optimal soil moisture. To reflect natural local cultivation conditions and minimize exogenous nutrient interference on fruit biochemistry, no supplemental fertilizers were applied during the growing season. The blueberry bushes were 5 years old, and fruit harvesting took place between June 6 and July 2, 2024, covering five distinctly defined maturity stages: Maturity Stage I (100% green surface), Maturity Stage II (25%–75% pink surface), Maturity Stage III (75%–100% purple surface), Maturity Stage IV (100% blue surface with prominent waxy bloom), and Maturity Stage V (dark blue, harvested 5 days after Stage IV). Maturity Stage IV represents the optimal commercial maturity characterized by full color development, while Maturity Stage V represents a late‐harvest (overripe) stage characterized by a duller appearance and noticeably reduced waxy bloom. The bushes underwent three replicate harvests at each maturity stage, with 300 fruits of consistent size and maturity (30 fruits per plant) randomly selected from 10 representative plants during each harvest. Subsequently, three replicate measurements were conducted for each maturity stage, with 50 fruits per replicate. To minimize the influence of external factors, all fruits were harvested on clear, sunny days between 9:00 and 11:00 AM. Immediately after harvest, fruits were transported to the laboratory in ventilated, cushioned containers within 2 h. Disease‐free fruits of uniform size and without obvious surface damage were selected as the experimental materials. All physicochemical and VOC analyses were initiated within 4 h of harvest to ensure the assessment of harvest‐quality attributes, thereby minimizing the impact of postharvest storage.

### 2.2 Reagent Materials and Instruments

The internal standard 3‐nonanone (99%) was obtained from Sigma‐Aldrich (Titan Co. Ltd., Shanghai, China) and prepared using a 10% methanol solution of high‐performance liquid chromatography‐grade methanol from Fisher Scientific (Pittsburgh, PA, USA). *n*‐Alkanes (C5–C25) standards were obtained from Anpel (Shanghai, China). Purified water was sourced from Wahaha Foods Co. Ltd. (Hangzhou, China), and sodium chloride (NaCl) was obtained from Macklin Co. Ltd. (Shanghai, China).

The JA5002 electronic balance was sourced from Jinghai Instrument Co. Ltd. (Shanghai, China). The 808 Titrando automatic potentiometric titrator was obtained from Metrohm (Herisau, Switzerland). The PR‐101α refractometer was obtained from ATAGO Corporation (Japan). The HWS‐26 electric heating constant temperature water bath was acquired from Yiheng Scientific Instrument Co. Ltd. (Shanghai, China). The TGL‐18 M desktop high‐speed refrigerated centrifuge was purchased from Luxiangyi Centrifuge Instrument Co. Ltd. (Shanghai, China). The T9CS + dual‐beam UV–visible spectrophotometer was obtained from Puxi General Instrument Co. Ltd. (Beijing, China). The LED Photography Box was sourced from Shentotem Technology Co. Ltd. (Shenzhen, China). The GCMS‐QP2020 single quadrupole gas chromatography–mass spectrometer was acquired from Shimadzu Corporation (Japan).

### 2.3 Measurement Indicators and Methods

#### 2.3.1 Sensory Quality

Fruit diameter (FD) and fruit length (FL) were assessed at five maturity stages using a digital caliper with a precision of 0.01 mm. The fruit shape index was determined as the ratio of FL to FD. Fresh fruit weight was measured using an electronic balance, and the individual fruit mass (g) was calculated by averaging the weight measurements.

Blueberry fruit firmness (200 g) was measured using a texture analyzer (Firmtech FT‐7, Germany) equipped with an 8‐mm‐diameter probe moving at a speed of 150 mm/min. Three experiments were performed on different sides of each blueberry.

#### 2.3.2 Physicochemical Index

The soluble solids content (SSC) was measured using a PR‐101α handheld refractometer from Japan. The titratable acid and vitamin C content were determined through acid–base titration and 2,6‐dichloroindophenol titration, respectively, using an 808 Titrando automatic potentiometric titrator provided by Metrohm. Each maturity stage was represented by three biological replicates, with 10 fruits analyzed per replicate. Soluble sugars were quantified using the BC003 plant soluble sugar content kit from Beijing Solaibao Technology Co. Ltd. (Beijing, China).

#### 2.3.3 Determination of VOCs

##### 2.3.3.1 Sample Preparation

Twenty fruits were randomly selected and evenly sampled, with 50 g of pulp weighed for each fruit. Subsequently, 18 g of NaCl was added into the pulp, and the mixture was crushed. The mixed sample (5 g) was then placed in a 20‐mL headspace bottle, to which 20 μL of a 0.04 g/L 3‐nonanone standard solution was added. The bottle was sealed with a polytetrafluoroethylene butyl synthetic rubber septum, and this process was repeated three times prior to taking measurements using GC–MS. The samples underwent VOCs extraction using a 50/30 μm DVB/CAR/PDMS extraction head (Supelco Company, USA) for 30 min at 60°C, followed by desorption for 3 min. Subsequently, aging was allowed for 10 min at 250°C.

##### 2.3.3.2 HS‐SPME Conditions

A 60 m × 0.25 mm × 0.25 μm HP‐INNOWAX column (Agilent Technologies, USA) was used together with a 50/30 μm DVB/CAR/PDMS extraction head. The polar stationary phase of this column (polyethylene glycol) was selected because it offers superior separation efficiency for oxygenated VOCs such as aldehydes, esters, alcohols, and ketones, which are the primary contributors to fruit aroma. This choice is consistent with methodologies commonly employed in the analysis of blueberry and other berry fruit VOCs (Pico et al. 2021; Li, Yang, et al. 2025). The temperature program included a sample inlet temperature of 250°C, a connection port temperature of 280°C, and a high‐purity helium carrier gas flowing at 1 mL/min without splitting the sample. The temperature was initially set at 30°C for 5 min, then increased to 210°C at the rate of 3°C/min and held for 5 min, followed by an increase to 250°C at the rate of 10°C/min for an additional 5 min.

##### 2.3.3.3 GC–MS Analysis

The samples were analyzed using a Shimadzu 201 Plus GC coupled with a QP2020 MS system from Shimadzu (Tokyo, Japan). To desorb the VOCs captured on the SPME fiber, the injector port was heated to 200°C, and desorption occurred for 1 min in splitless mode. The column temperature was initially set at 40°C for 1 min, then increased to 200°C at a rate of 2°C/min and held for 1 min. Subsequently, the temperature was ramped up to 230°C at a rate of 10°C/min and held for 10 min. Helium of 99.999% purity served as the carrier gas at a constant flow rate of 1 mL/min.

MS analysis was performed in electron ionization (EI) mode with an ionization energy of 70 eV. The ion source of the detector was maintained at 230°C throughout the analysis, and mass spectra were acquired across the 50–500 m/z range.

##### 2.3.3.4 Semi‐Quantification of VOCs

Qualitative analysis was based on a similarity search after integration of all ion chromatographic peaks by the reanalysis module of the Agilent qualitative analysis software version b.07.00. VOCs with a ≥ 90% match were screened out, and their retention indices (RIs) were determined according to the retention times of *n*‐alkanes (C5–C25) standards (Anpel, Shanghai, China), followed by a comparison of their RI values with reference values in the NIST 17 database (Guo et al. 2022). Semi‐quantification of VOCs in blueberry was based on the linear relationship between peak areas and concentrations of the internal standard (3‐nonanone) and VOCs, following the literature with slight modifications (Wang et al. 2024).

#### 2.3.4 Sensory Evaluation

The fruit flavor assessment panel comprised 10 skilled members (five males and five females) aged 22–29 years. Following the guidelines outlined in the national standards GB/T 10221‐2021 and GB/T 12315‐2021 for sensory evaluation in China, the evaluators assessed the aroma of blueberry samples and performed a quantitative descriptive analysis. The evaluation was conducted in a controlled environment at 24°C ± 2°C and 55% humidity. The panelists identified and rated nine oral sensations (crispness, juiciness, sourness, sweetness, astringency, green, fruity, floral, fragrant) to characterize the sensory attributes of the blueberry sample (Bartoshuk et al. 2003; Zheng et al. 2025). The reference standard is provided in Table S1. The panelists rinsed their palates with purified water between samples. Each attribute's intensity was evaluated on an 11‐point scale ranging from 0 (lowest) to 10 (highest). The samples were presented in a randomized sequence across three replicates.

Ethical approval for the sensory evaluation was not necessary according to the institutional policy of the Institute of Pomology, Chinese Academy of Agricultural Sciences. All participants provided written informed consent prior to the evaluation. Furthermore, the rights and privacy of all participants were protected throughout the study, which included no coercion to participate, full disclosure of the procedures, and the freedom to withdraw at any time.

### 2.4 Odor Activity Value (OAV)

The OAV serves as a metric for assessing the impact of aromatic compounds in food on the overall aroma profile. It is determined by comparing the concentration of these compounds with their respective odor thresholds using the following formula: OAV = concentration of a VOC/its odor threshold. Aroma components with OAV values exceeding 1 are deemed to have a substantial influence on food aroma, whereas those with OAV values between 0.1 and 1 are considered to play a modifying role in flavor perception. The odor thresholds used for OAV calculations were primarily sourced from the established literature on fruit and food aroma compounds. The thresholds for the major aldehyde and alcohol compounds and aroma characteristics were adopted from previous comprehensive studies (Sun et al. 2024; Wang et al. 2024; Toledo Guerrero et al. 2024). Additional references that have been widely cited in fruit aroma research were consulted for specific compounds where necessary (Leffingwell and Leffingwell 1991; Wang et al. 2008; Van Gemert 2011; Sun and Chen 2016).

### 2.5 Data Processing and Statistical Analysis

Data analysis and visualization were carried out using Excel 2019 (Microsoft, Redmond, WA, USA). Orthogonal partial least squares discriminant analysis (OPLS‐DA) and variable importance in projection (VIP) calculations were performed in SIMCA 14.1 (Umetrics, Umeå, Sweden). To evaluate differences in VOC concentrations among the five maturity stages, one‐way analysis of variance (ANOVA) was conducted, and Tukey's honestly significant difference (HSD) post hoc test was applied for multiple comparisons. Prior to ANOVA, data were examined for normality and homogeneity of variance, and log‐normalized when necessary. All statistical tests were two‐tailed, and statistical significance was defined as *p* < 0.05. In the tables, values sharing the same superscript letter are not significantly different, whereas different letters indicate significant differences at *p* < 0.05. Correlation analysis and hierarchical cluster analysis were performed using SPSS 22.0 (IBM Corp., Armonk, NY, USA), and the corresponding figures (heatmaps/dendrograms) were generated in Origin 2024 (OriginLab, Northampton, MA, USA).

## 3 Results

### 3.1 Sensory Analysis

**FIGURE 1:**
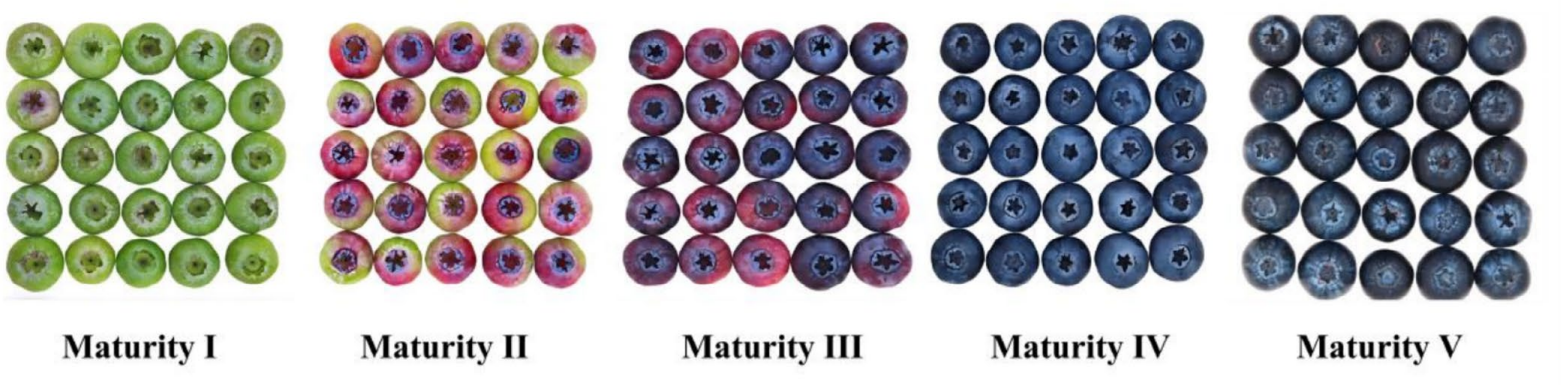
Illustration of “Legacy” blueberry at various maturity stages.

Distinct visual and sensory characteristics were observed at different blueberry maturity stages (Figure 1). The detailed sensory scores from the trained panel are provided in Table S2. Maturity Stage I blueberries exhibited a predominant green color, a lack of skin luster, a firm texture, a lack of elasticity upon pressure, and a sour, sharp taste. In contrast, Maturity Stage II fruit displayed a combination of light purple or pink hues, a firm texture, and a subtle to negligible fruity aroma. Maturity Stage III fruit demonstrated a blend of purple‐red and deep purple colors, slightly tough skin, loose flesh, pronounced acidity with initial sweetness, a faint bitter aftertaste, and a mild fruity aroma. Maturity Stage IV fruit was predominantly deep purple or blue, displayed a natural wax coating, and boasted a warm luster, full and round texture, slight pressure rebound, high sweetness, soft acidity, and a rich berry fruit aroma. Maturity Stage V fruit appeared dark purple or black‐brown, devoid of fruit powder, with soft, easily malleable flesh. It presented a sweet, dense, and fermented taste with minimal acidity.

### 3.2 Analysis of Blueberry Maturity Stages

Table 1 demonstrates that blueberry fruit diameter and length increased significantly as maturity progressed, whereas the fruit shape index remained constant across all maturity stages. The fresh fruit weight peaked at Maturity Stage V (2.41 g), significantly surpassing weights at other maturity stages. Fruit firmness decreased progressively during ripening, with the highest value observed at Maturity Stage I (408.41 g/mm). The SSC showed a positive correlation with maturity, being lowest in Maturity Stage I fruit (7.7%) and highest in Maturity Stage IV fruit (14.66%). No significant difference in the SSC was found between Maturity Stage IV and V fruits, indicating a stabilization of solids accumulation after maturation. In contrast, the soluble sugar content increased notably throughout maturation, rising from 7.427 to 80.44 mg/100 g in Maturity Stage I and V, respectively. The vitamin C content also increased with maturity, being lowest in Maturity Stage I (4.165 mg/100 g). Maturity Stage III to V fruits had significantly higher vitamin C levels than earlier maturity stage fruits (*p* < 0.05).

**TABLE 1:** Effects of different maturity stages on blueberry quality.

Index	Maturity				
	I	II	III	IV	V
Fresh fruit weight (g)	1.008 ± 0.15^d^	1.328 ± 0.13^c^	1.474 ± 0.14^c^	1.96 ± 0.27^b^	2.412 ± 0.43^a^
Firmness (g/mm)	408.41 ± 0.23^a^	282.77 ± 0.53^b^	242.67 ± 0.42^b^	176.82 ± 0.57^c^	169.06 ± 0.61^c^
Fruit transverse diameter (mm)	12.87 ± 0.43^c^	14.44 ± 0.32^b^	14.67 ± 0.79^b^	15.63 ± 0.78^ab^	16.12 ± 1.02^a^
Fruit longitudinal diameter (mm)	9.63 ± 0.41^b^	10.55 ± 0.29^a^	11.05 ± 0.41^a^	11.5 ± 0.53^a^	11.6 ± 0.55^a^
Fruit shape index	1.338 ± 0.04^a^	1.37 ± 0.05^a^	1.327 ± 0.04^a^	1.359 ± 0.53^a^	1.39 ± 0.14^a^
SSC (%)	7.7 ± 0.67^c^	10.81 ± 0.69^b^	11.97 ± 0.49^b^	14.66 ± 0.79^a^	14.65 ± 0.74^a^
TA (%)	3.608 ± 0.19^a^	1.896 ± 0.14^b^	1.755 ± 0.16^b^	0.615 ± 0.09^c^	0.436 ± 0.06^c^
SS (mg·100 g^−1^)	7.427 ± 0.27^d^	42.51 ± 0.68^c^	46.72 ± 0.79^c^	67.58 ± 1.28^b^	80.44 ± 2.23^a^
VC (mg·100 g^−1^)	4.165 ± 0.03^c^	5.7287 ± 0.08^b^	7.159 ± 0.21^a^	7.824 ± 0.14^a^	7.04 ± 0.03^a^

*Note:* The different lowercase letters (a–d) indicate significant differences among treatment groups (*p* < 0.05). Values are mean ± SD (*n* = 10 per stage).

### 3.3 Analysis of VOCs in Blueberry at Different Maturity Stages

**FIGURE 2:**
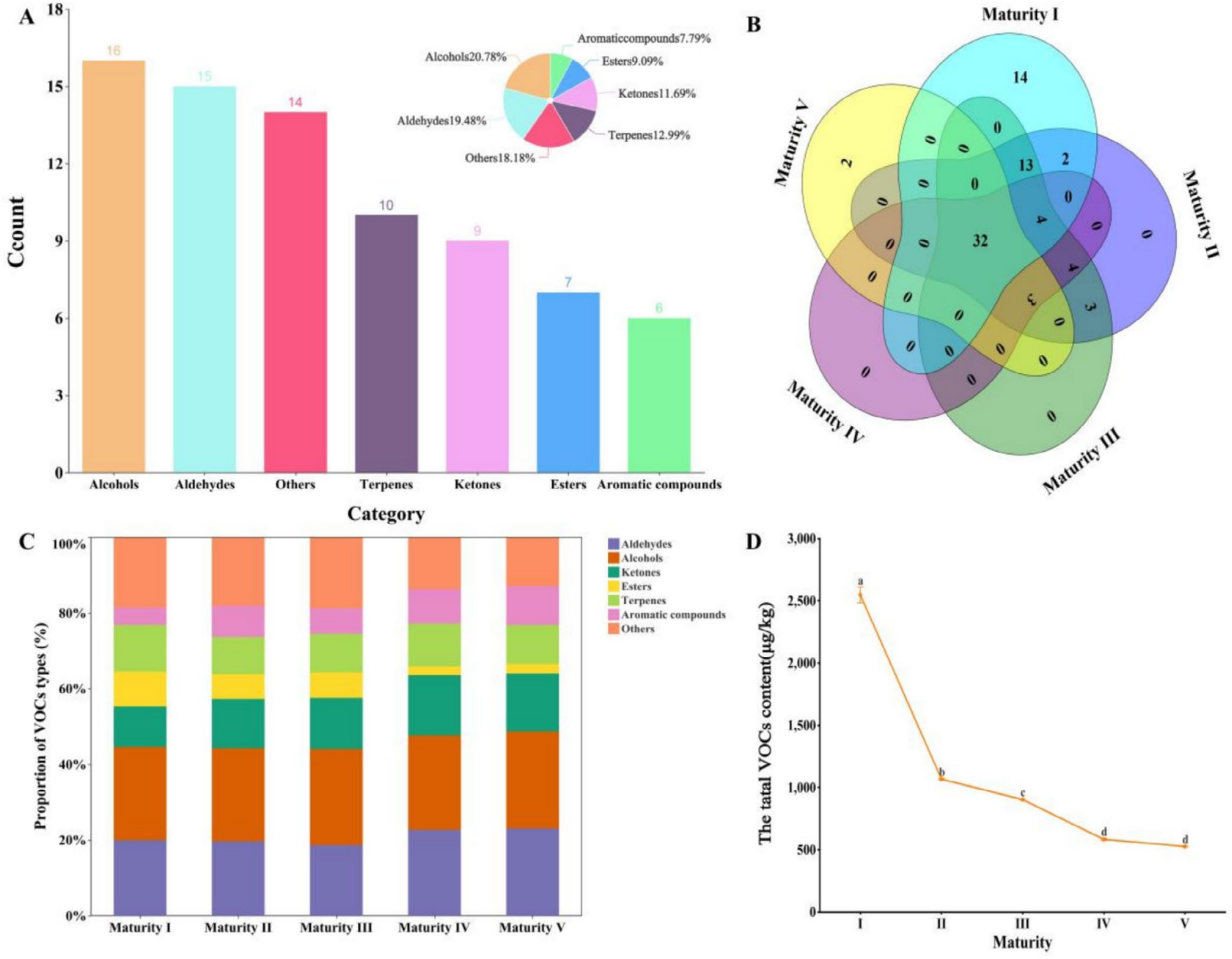
Composite histogram of VOC types (A), Venn diagram (B), and composition of VOCs in blueberry at different maturity stages (C). Line chart of total VOCs (D). Different letters above points indicate significant differences among stages (*p* < 0.05).

HS‐SPME‐GC–MS detected 77 VOCs in blueberries at different maturity stages (Table 2). These compounds included 15 aldehydes, 16 alcohols, nine ketones, seven esters, 10 terpenes, six aromatic compounds, and 14 miscellaneous compounds. Aldehydes and alcohols were the predominant classes of VOCs, collectively constituting over 80% of the total VOCs (Figure 2A–C). Aldehydes were consistently the most abundant VOC class across all maturity stages, representing 59.09%, 73.69%, 69.96%, 85.18%, and 81.67% of the total detected VOCs in Maturity Stages I to V, respectively. The abundance of total VOCs was highest in Maturity Stage I, followed by Maturity Stages II and III, and lowest in Maturity Stages IV and V as illustrated in Figure 2D.

**TABLE 2:** Composition and content of volatile organic compounds (VOCs) in blueberries at different maturity stages.

Number	Category	Compounds	CAS Number	Content (μg/kg FW)				
				Maturity I	Maturity II	Maturity III	Maturity IV	Maturity V
1	Alcohols	1‐Hexanol	111‐27‐3	20.21 ± 5.23ᵃ	13.43 ± 5.69ᵃᵇ	7.30 ± 1.57ᵇ	2.46 ± 0.33ᶜ	6.78 ± 2.67ᵇ
2		1‐Hexanol, 2‐ethyl—	104‐76‐7	4.71 ± 1.23ᵃ	3.06 ± 0.35ᵃᵇ	3.51 ± 1.28ᵃ	2.64 ± 0.54ᵃᵇ	2.55 ± 0.17ᵇ
3		1‐Nonanol	143‐8‐8	1.59 ± 0.5ᵃ	0.40 ± 0.02ᵇ	0.62 ± 0.017ᵇ	—	—
4		1‐Octanol	111‐87‐5	1.08 ± 0.2ᵃᵇ	0.50 ± 0.06ᵇ	0.53 ± 0.06ᵇ	0.86 ± 0.2ᵃᵇ	1.33 ± 0.34ᵃ
5		1‐Octen‐3‐ol	3391‐86‐4	2.46 ± 0.37ᵃ	2.16 ± 0.18ᵃ	1.40 ± 0.13ᵇ	—	—
6		1‐Pentanol	71‐41‐0	9.60 ± 4.37ᵃ	7.20 ± 4.28ᵃᵇ	4.00 ± 0.5ᵇ	4.46 ± 1.45ᵇ	4.79 ± 0.68ᵇ
7		1‐Penten‐3‐ol	616‐25‐1	44.86 ± 8.58ᵃ	6.49 ± 2.56ᵇ	6.57 ± 3.41ᵇ	3.06 ± 1.06ᵇ	4.28 ± 1.46ᵇ
8		2,6‐Octadien‐1‐ol, 3,7‐dimethyl‐, (Z)—	106‐25‐2	4.53 ± 0.54ᵃ	1.73 ± 0.52ᵇ	2.37 ± 0.05ᵃᵇ	0.66 ± 0.09ᶜ	2.38 ± 0.98ᵃᵇ
9		2‐Hexen‐1‐ol, (E)—	928‐95‐0	75.15 ± 17.83ᵃ	61.52 ± 7.68ᵃ	46.51 ± 11.27ᵃ	6.82 ± 1.32ᵇ	14.53 ± 4.72ᵇ
10		2‐Hexen‐1‐ol, acetate, (Z)—	56922‐75‐9	4.60 ± 1.47ᵃ	5.84 ± 0.34ᵃ	3.16 ± 0.04ᵇ	—	—
11		2‐Methyl‐6‐hepten‐3‐ol	78631‐45‐5	3.37 ± 0.71ᵃ	—	—	—	—
12		2‐Penten‐1‐ol, (Z)—	1576‐95‐0	27.30 ± 9.35ᵃ	6.82 ± 1.62ᵇ	7.56 ± 4.52ᵇ	2.45 ± 0.51ᵇ	2.01 ± 0.61ᵇ
13		3‐Buten‐2‐ol, 2‐methyl—	115‐18‐4	2.46 ± 0.47ᵃ	1.94 ± 0.19ᵃ	1.79 ± 0.61ᵃ	—	—
14		3‐Hexen‐1‐ol	544‐12‐7	405.42 ± 25.92ᵃ	23.84 ± 4.39ᵇ	24.77 ± 8.51ᵇ	2.52 ± 0.53ᶜ	5.64 ± 1.27ᶜ
15		Eucalyptol	470‐82‐6	14.51 ± 4.24ᵃ	3.75 ± 0.79ᵇ	2.43 ± 0.74ᵇ	1.44 ± 0.18ᵇ	—
16		L‐alpha‐Terpineol	10482‐56‐1	16.88 ± 6.71ᵃ	12.08 ± 3.52ᵃᵇ	8.87 ± 2.78ᵇ	1.74 ± 0.42ᶜ	—
17	Aldehydes	2,4‐Hexadienal, (E,E)—	142‐83‐6	11.71 ± 4.36ᵃ	3.81 ± 2.14ᵇ	3.57 ± 0.13ᵇ	1.40 ± 0.21ᵇ	—
18		2‐Heptenal, (E)—	18829‐55‐5	9.55 ± 3.56ᵃ	7.54 ± 2.45ᵃᵇ	3.91 ± 1.33ᵇ	1.61 ± 0.83ᵇ	1.35 ± 0.25ᵇ
19		2‐Hexenal, (E)—	6728‐26‐3	1292.81 ± 223.52ᵃ	676.18 ± 22.34ᵇ	587.12 ± 30.25ᵇ	433.84 ± 82.55ᶜ	343.84 ± 19.54ᶜ
20		2‐Nonenal, (E)—	18829‐56‐6	0.85 ± 0.82ᵃ	—	—	—	—
21		2‐Octenal, (E)—	2548‐87‐0	4.85 ± 2.52ᵃ	3.50 ± 1.25ᵃ	1.79 ± 0.31ᵃ	—	—
22		2‐Pentenal, (E)—	1576‐87‐0	32.89 ± 8.1ᵃ	7.16 ± 0.93ᵇ	7.23 ± 1.76ᵇ	1.89 ± 0.55ᶜ	1.88 ± 0.31ᶜ
23		3‐Hexenal	4440‐65‐7	29.29 ± 9.61ᵃ	4.67 ± 1.5ᵇ	4.27 ± 1.25ᵇ	2.04 ± 0.42ᵇ	1.23 ± 0.24ᵇ
24		Butanal	123‐72‐8	0.74 ± 0.22ᵃ	0.47 ± 0.08ᵃᵇ	0.37 ± 0.07ᵇ	0.44 ± 0.23ᵃᵇ	—
25		Decanal	112‐31‐2	14.40 ± 4.22ᵃ	4.52 ± 1.32ᵇ	—	—	—
26		Heptanal	111‐71‐7	—	—	—	—	1.89 ± 0.23ᵃ
27		Hexanal	66‐25‐1	62.19 ± 17.54ᵃ	38.83 ± 4.96ᵇ	25.17 ± 5.78ᵇ	39.82 ± 5.31ᵇ	64.71 ± 15.61ᵃ
28		Nonanal	124‐19‐6	13.74 ± 6.14ᵃ	4.70 ± 0.98ᵇ	3.89 ± 0.61ᵇ	7.71 ± 3.31ᵃᵇ	9.22 ± 2.81ᵃᵇ
29		Octanal	124‐13‐0	5.02 ± 2.13ᵃ	1.60 ± 0.16ᵇ	1.76 ± 0.54ᵇ	2.15 ± 0.77ᵃᵇ	2.01 ± 0.43ᵃᵇ
30		Pentanal	110‐62‐3	—	9.74 ± 1.98ᵃ	7.42 ± 3.34ᵃ	4.64 ± 1.72ᵃ	5.79 ± 1.24ᵃ
31		Pentanal, 2‐methyl—	123‐15‐9	9.19 ± 1.52ᵃ	—	—	—	—
32	Terpenes	alpha‐Farnesene	502‐61‐4	5.43 ± 2.07ᵃ	—	—	—	—
33		beta‐Myrcene	123‐35‐3	10.03 ± 5.61ᵃ	2.70 ± 0.75ᵇ	3.60 ± 1.04ᵇ	3.15 ± 0.93ᵇ	2.43 ± 0.61ᵇ
34		beta‐Ocimene	13877‐91‐3	4.33 ± 1.75ᵃ	1.67 ± 0.37ᵇ	2.27 ± 0.56ᵃᵇ	1.51 ± 0.45ᵇ	0.98 ± 0.02ᵇ
35		1,3‐Pentadiene, (E)—	2004‐70‐8	2.21 ± 0.86ᵃ	—	—	—	—
36		1,5,7‐Octatrien‐3‐ol, 3,7‐dimethyl—	29957‐43‐5	—	11.23 ± 0.41ᵃ	6.20 ± 3.07ᵃ	0.67 ± 0.03ᵇ	—
37		2,4,6‐Trimethyl‐1,3,6‐heptatriene	0‐0‐0	—	1.37 ± 0.07ᵃ	1.72 ± 0.11ᵃ	—	—
38		D‐Limonene	5989‐27‐5	12.90 ± 7.62ᵃ	5.10 ± 0.34ᵇ	6.13 ± 2.31ᵇ	1.75 ± 0.72ᶜ	3.08 ± 0.46ᵇᶜ
39		Humulene	6753‐98‐6	12.94 ± 5.36ᵃ	—	—	—	—
40		Isoprene	78‐79‐5	0.85 ± 0.02ᵃ	—	—	—	—
41		Linalool	78‐70‐6	49.26 ± 11.59ᵃ	28.12 ± 14.28ᵃᵇ	21.77 ± 6.23ᵇ	14.61 ± 3.14ᵇ	14.21 ± 6.82ᵇ
42	Ketones	1‐Octen‐3‐one	4312‐99‐6	—	1.41 ± 0.85ᵃ	2.36 ± 0.63ᵃ	0.95 ± 0.17ᵃ	—
43		2,3‐Octanedione	585‐25‐1	—	1.39 ± 0.44ᵃ	2.41 ± 0.67ᵃ	0.85 ± 0.15ᵃ	0.73 ± 0.05ᵃ
44		2‐Heptanone	110‐43‐0	8.24 ± 3.61ᵃ	2.31 ± 0.71ᵇ	2.21 ± 0.94ᵇ	0.94 ± 0.09ᵇ	1.17 ± 0.19ᵇ
45		2‐Heptanone, 6‐methyl—	928‐68‐7	1.48 ± 0.17ᵃ	0.56 ± 0.14ᵇ	0.57 ± 0.14ᵇ	—	—
46		2‐Nonanone	821‐55‐6	28.74 ± 4.53ᵃ	8.67 ± 2.16ᵇ	8.74 ± 3.13ᵇ	2.56 ± 0.64ᶜ	2.78 ± 0.26ᶜ
47		2‐Undecanone	112‐12‐9	10.38 ± 2.15ᵃ	3.51 ± 0.78ᵇ	4.14 ± 0.74ᵇ	1.20 ± 0.04ᶜ	1.16 ± 0.33ᶜ
48		3‐Pentanone	96‐22‐0	5.74 ± 1.04ᵃ	—	—	—	—
49		5,9‐Undecadien‐2‐one, 6,10‐dimethyl‐, (E)—	3796‐70‐1	3.16 ± 0.97ᵃ	3.29 ± 0.29ᵃ	4.67 ± 1.79ᵃ	1.53 ± 0.38ᵃ	1.44 ± 0.51ᵃ
50		5‐Hepten‐2‐one, 6‐methyl—	110‐93‐0	41.62 ± 16.19ᵃ	23.69 ± 7.19ᵃ	28.29 ± 5.63ᵃ	10.15 ± 5.24ᵃ	11.13 ± 6.1ᵃ
51	Esters	2,2,4‐Trimethyl‐1,3‐pentanediol diisobutyrate	6846‐50‐0	4.75 ± 1.14ᵃ	1.62 ± 0.53ᵇ	2.06 ± 0.78ᵇ	1.19 ± 0.25ᵇ	0.81 ± 0.06ᵇ
52		3‐Hexen‐1‐ol, acetate, (E)—	3681‐82‐1	64.26 ± 24.24ᵃ	—	—	—	—
53		Acetic acid, methyl ester	79‐20‐9	1.23 ± 0.09ᵃ	0.87 ± 0.13ᵇ	0.48 ± 0.04ᶜ	—	—
54		Butanoic acid, 2‐ethyl‐, 1,2,3‐propanetriyl ester	0‐0‐0	0.90 ± 0.06ᵃ	0.63 ± 0.12ᵃ	1.19 ± 0.34ᵃ	—	—
55		Butanoic acid, 3‐hexenyl ester, (Z)—	16491‐36‐4	7.52 ± 3.21ᵃ	—	—	—	—
56		Methyl valerate	624‐24‐8	—	1.54 ± 0.57ᵃ	2.47 ± 0.67ᵃ	—	—
57		Propanoic acid, 2‐penten‐1‐yl ester (Z)—	0‐0‐0	1.09 ± 0.5ᵃ	—	—	—	—
58	Aromatic compounds	Benzene, 1,3‐dimethyl—	108‐38‐3	1.86 ± 0.34ᵃ	0.88 ± 0.08ᵇ	1.16 ± 0.27ᵃᵇ	1.04 ± 0.22ᵃᵇ	1.09 ± 0.44ᵃᵇ
59		Benzene, 1‐methyl‐3‐(1‐methylethenyl)—	1124‐20‐5	2.76 ± 1.26ᵃ	0.65 ± 0.17ᵇ	—	—	—
60		Benzyl alcohol	100‐51‐6	—	—	—	—	0.68 ± 0.34ᵃ
61		Ethylbenzene	100‐41‐4	—	0.56 ± 0.12ᵃ	0.66 ± 0.09ᵃ	0.38 ± 0.01ᵃ	—
62		o‐Xylene	95‐47‐6	—	1.42 ± 0.49ᵃ	1.83 ± 0.92ᵃ	1.38 ± 0.07ᵃ	1.06 ± 0.32ᵃ
63		Toluene	108‐88‐3	5.14 ± 2.22ᵃ	4.89 ± 1.23ᵃ	3.77 ± 1.47^b^	3.31 ± 0.72^c^	4.00 ± 2.63^b^
64	Others	3‐Hexenoic acid, (E)—	1577‐18‐0	8.17 ± 4.69ᵃ	—	—	—	—
65		Butanoic acid, 2,2‐dimethyl—	595‐37‐9	2.72 ± 0.49ᵃ	2.21 ± 0.6ᵃ	2.35 ± 1.2ᵃ	1.79 ± 0.27ᵃ	1.53 ± 0.19ᵃ
66		Butanoic acid, 2‐methyl—	116‐53‐0	2.16 ± 0.88ᵃ	0.61 ± 0.13ᵇ	0.60 ± 0.08ᵇ	—	—
67		Decane	124‐18‐5	—	0.34 ± 0.02ᵃ	0.33 ± 0.07ᵃ	0.41 ± 0.09ᵃ	—
68		Furan, 2‐ethyl—	3208‐16‐0	57.89 ± 6.22ᵃ	9.81 ± 3.92ᵇ	8.01 ± 2.45ᵇ	—	—
69		Furan, 2‐methyl—	534‐22‐5	0.70 ± 0.09ᵃ	0.56 ± 0.09ᵃ	0.34 ± 0.08ᵇ	—	—
70		Furan, 2‐pentyl—	3777‐69‐3	1.49 ± 0.14ᵃ	0.79 ± 0.17ᵇ	0.37 ± 0.15ᶜ	—	—
71		Furan, tetrahydro‐3‐methyl—	13423‐15‐9	0.82 ± 0.27ᵃ	0.76 ± 0.08ᵃ	0.43 ± 0.03ᵇ	0.68 ± 0.15ᵃ	0.56 ± 0.1ᵃᵇ
72		Hexanoic acid	142‐62‐1	6.27 ± 2.34ᵃ	5.58 ± 1.72ᵃ	5.10 ± 2.13ᵃ	1.89 ± 0.46ᵇ	1.40 ± 0.18ᵇ
73		Nonane	111‐84‐2	1.59 ± 0.06ᵃ	—	—	—	—
74		Octanoic acid	124‐07‐2	—	1.43 ± 0.11ᵃ	1.87 ± 0.65ᵃ	—	—
75		Propanoic acid	1979‐09‐04	1.61 ± 0.22ᵃ	1.67 ± 0.13ᵃ	1.44 ± 0.41ᵃ	2.66 ± 0.36ᵃ	1.05 ± 0.31ᵃ
76		trans‐2‐(2‐Pentenyl)furan	70424‐14‐5	6.90 ± 1.68ᵃ	—	—	—	—

*Note:* The different lowercase letters (a–c) indicate significant differences among treatment groups (*p* < 0.05). Values are mean ± SD (*n* = 3 per stage). “‐” Indicates not detected, the table below is the same.

**FIGURE 3:**
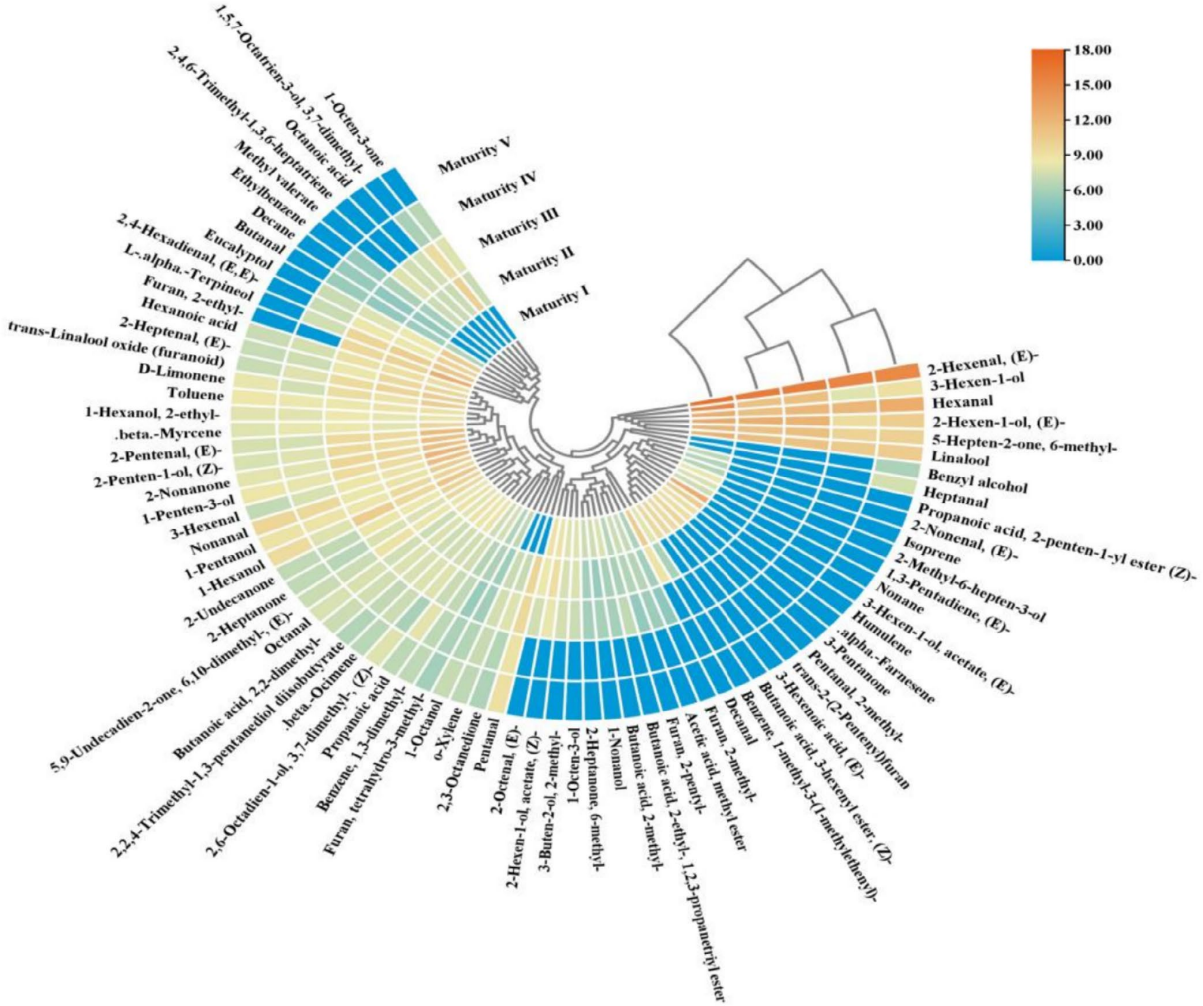
Clustering heat map of VOCs in blueberry at different maturity stages. Rows represent VOCs and columns represent stages. Color scale indicates log‐normalized intensity.

Cluster analysis of VOCs revealed distinct patterns among blueberries at different maturity stages, initially segregating into two main branches and subsequently forming three distinct groups (Figure 3). Maturity Stage I blueberries, containing 65 VOCs, displayed notably elevated concentrations of alcohols, mainly (*E*)‐2‐hexen‐1‐ol, 3‐hexen‐1‐ol, linalool, 1‐penten‐3‐ol, 2‐penten‐1‐ol, 1‐hexanol, and eucalyptol, compared with other maturity stages. The peak levels of 3‐hexen‐1‐ol (405.42 μg/kg) and (*E*)‐2‐hexenal (1292.81 μg/kg) were particularly high at Maturity Stage I. Maturity Stage II and III fruits contained 61 and 59 VOCs, respectively, demonstrating no significant difference in the total VOC content. However, substantial VOC compositional changes were observed across the stages, characterized by a decline or absence of terpenes (e.g., humulene, (*E*)‐1,3‐pentadiene, isoprene, α‐farnesene) and the emergence of new compounds such as 2,3‐octanedione, 1‐octen‐3‐one, pentanal, and methyl valerate. By Maturity Stage IV, the VOC diversity decreased to 44 compounds, with the content of acidic compounds diminishing to undetectable levels. Maturity Stage V blueberries exhibited a further reduction to 38 VOCs, with slightly lower concentrations than Maturity Stage IV blueberries. Later maturity stages revealed new VOCs such as heptanal and benzyl alcohol.

### 3.4 Differential Volatile Metabolites

**FIGURE 4:**
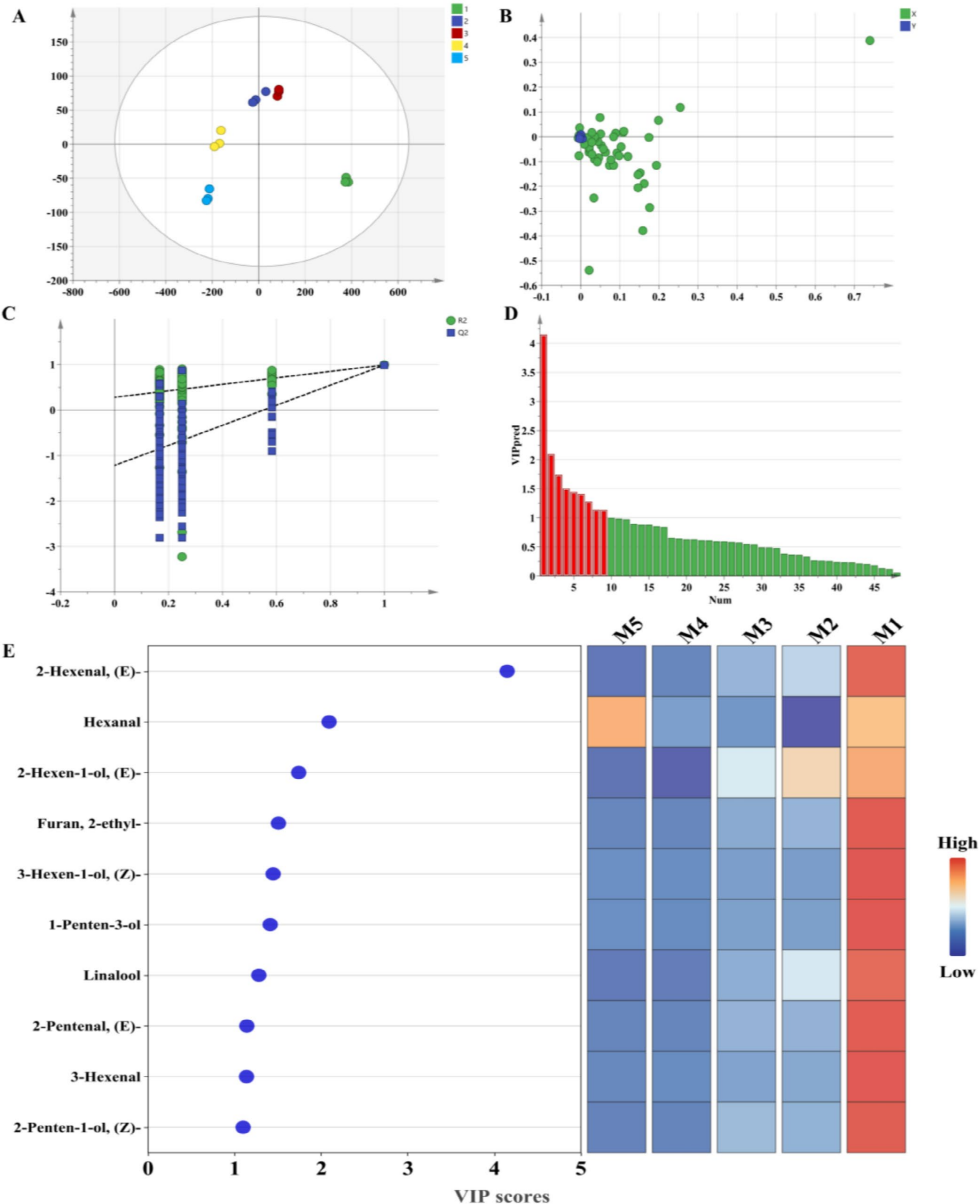
Differential analysis of VOCs in blueberry at different maturity stages based on orthogonal partial least squares discrimination analysis. Score scatter plot (A), data load scatter plot (B), model cross validation results (C), variable importance in projection (VIP) value (D), and dot bar heat map (E).

OPLS‐DA is a supervised statistical method used for discriminant analysis. It establishes a model to relate target objects to sample categories and is commonly applied in the analysis of flavor volatiles for sample categorization prediction (Wu et al. 2022; Shin et al. 2024). The model exhibited high fitting indices for both independent (Rx^2^ = 0.988) and dependent variables (Ry^2^ = 0.860), as well as a model prediction index (Q^2^) of 0.848, all exceeding 0.5 (Figure 4A,B). A steeper slope and lower intercept indicate a more robust model with enhanced quality and predictive capacity. Through 200 permutation tests (Figure 4C), the *Q* ^2^ regression line intersected the *y*‐axis below zero, suggesting the model's reliability. The VIP score, representing the weight of OPLS‐DA model variables, quantifies the influence and explanatory power of each component on sample classification and discrimination. A VIP value exceeding 1.0 (Figure 4D) is widely used as a threshold to identify the most relevant compounds responsible for group separation, as these variables have an above‐average influence on the model (Zhang et al. 2024). OPLS‐DA identified 10 differential volatile metabolites that effectively distinguished between the five maturity stages (Figure 4E): (*E*)‐2‐hexenal, hexanal, (*E*)‐2‐hexen‐1‐ol, 2‐ethyl‐furan, 3‐hexen‐1‐ol, 1‐penten‐3‐ol, linalool, (*E*)‐2‐pentenal, 3‐hexenal, and 2‐penten‐1‐ol.

### 3.5 Key Aroma Components of Blueberry

Table 3 lists 17 key aroma components identified based on odor activity values (OAV > 1). These include (*E*)‐2‐hexenal, (*E*)‐2‐nonenal, (*E*)‐2‐octenal, butanal, decanal, hexanal, nonanal, octanal, pentanal, 2‐methyl‐pentanal, 1‐octanol, 3‐hexen‐1‐ol, 1‐octen‐3‐one, 2‐nonanone, β‐myrcene, humulene, and (*E*)‐3‐hexenoic acid. (*E*)‐2‐hexenal exhibited OAVs greater than 1 at all maturity stages, serving as the foundational aroma component throughout ripening. The composition of these key volatiles shifted significantly across stages. During Maturity Stage I, the profile was dominated by floral, grassy, and green‐leaf aroma compounds, including humulene, (*E*)‐3‐hexenoic acid, 2‐methylpentanal, and 3‐hexen‐1‐ol. As maturation progressed from Maturity Stages II to IV, 1‐octen‐3‐one, characterized by earthy and mushroom notes, became present at significant concentrations. Additionally, β‐myrcene, providing sweet and creamy undertones, remained consistently present across all maturity stages. Late maturity stages (Maturity Stages IV and V) were specifically enriched in aldehydes like hexanal, nonanal, and octanal, which are associated with citrus and floral notes.

**TABLE 3:** Odor activity values (OAVs) and aroma characteristics of the principal VOCs in blueberries at different maturity stages.

Compound	Aroma Character	Oder Threshold/(μg/kg)	OAV value				
2‐Hexenal, (E)—	Fruit and green leaf aroma	88.50	14.61	7.64	6.63	4.90	3.89
2‐Nonenal, (E)—	Cucumber aroma	0.15	5.64	—	—	—	—
2‐Octenal, (E)—	The aroma of oranges and hazelnuts	3.00	1.62	1.17	0.60	—	—
Butanal	Acrid odor	0.67	1.10	0.70	0.56	0.66	—
Decanal	Citrus, wax, and floral aromas	4.90	2.94	0.92	—	—	—
Hexanal	Oil and grass aroma	4.50	13.82	8.63	5.59	8.85	14.38
Nonanal	Rose and citrus aromas	1.10	12.49	4.27	3.54	7.01	8.39
Octanal	Fat wax fragrance, with fruity jasmine aroma	0.80	6.28	2.00	2.20	2.69	2.52
Pentanal	Apple and strawberry fragrance	0.41	—	23.74	18.09	11.32	14.13
Pentanal, 2‐methyl—	Ether aroma, green aroma and fruit aroma	1.60	5.74	—	—	—	—
1‐Octanol	Oil and citrus aroma	0.80	1.35	0.62	0.67	1.08	1.66
3‐Hexen‐1‐ol	Green tender leaves with a fragrant aroma	70	5.79	0.34	0.35	0.04	0.08
1‐Octen‐3‐one	Fresh soil, mushroom, vegetable aroma	0.01	—	140.86	235.92	95.20	—
2‐Nonanone	Cream and fruit flavors	8.20	3.50	1.06	1.07	0.31	0.34
Beta‐Myrcene	Pepper fragrance, sweet and creamy aroma	1.20	8.36	2.25	3.00	2.63	2.03
Humulene	A faint aroma of cloves	0.01	1470.91	—	—	—	—
3‐Hexenoic acid, (E)—	Sweet green fruit, jackfruit, green fragrance	0.07	121.95	—	—	—	—

### 3.6 Pearson's Correlation Coefficient Analysis

**FIGURE 5:**
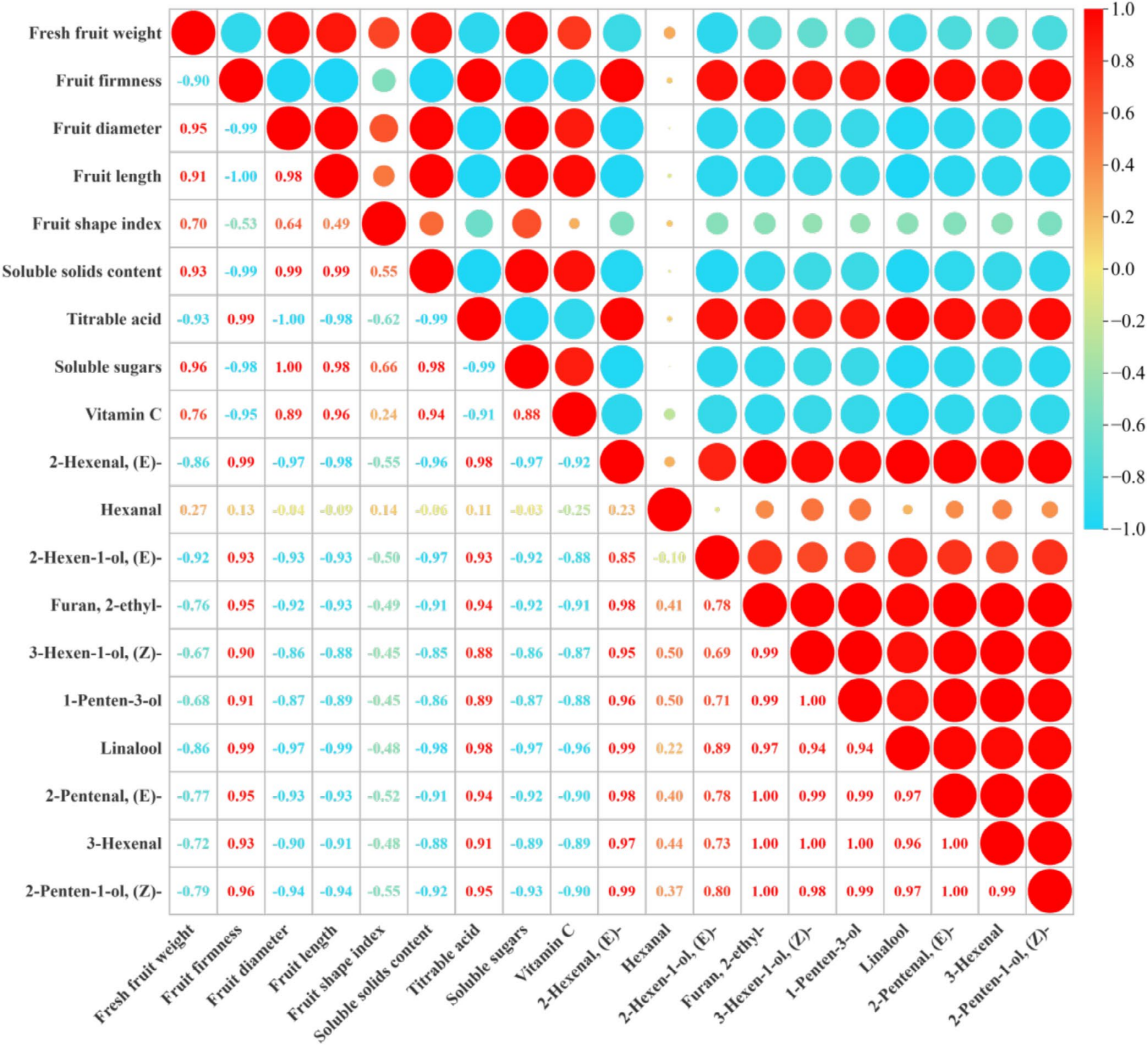
Pearson correlation analysis between fruit quality attributes and VIP value (VIP > 1). Red and blue colors represent significant (*p* < 0.05) positive and negative correlations between variables, respectively. The size of the circle indicates the strength of the correlation coefficient, with a larger circle indicating a stronger correlation between variables.

The relationship between fruit quality and 10 odor‐active substances (OAV > 1) was assessed based on fruit maturity stages (Figure 5). Fruit firmness exhibited a significant positive correlation with titratable acidity (TA) and negative correlations with SSC, vitamin C, and soluble sugars. Conversely, the fresh fruit weight displayed inverse relationships with these attributes. Volatile substances with VIP scores exceeding 1 were positively associated with fruit firmness and TA but showed negative correlations with other physicochemical parameters. Notably, hexanal exhibited weak or nonsignificant correlations with most high‐VIP VOCs.

## 4 Discussion

### 4.1 Evolution of Physicochemical and Sensory Properties During Maturation

Blueberry ripening is a multifaceted process characterized by continuous changes in skin color, fruit texture, and nutrient accumulation. The analysis revealed a progressive decrease in fruit firmness, aligning with patterns observed in other berry fruits (Toledo Guerrero et al. 2024). Concurrently, individual fruit weight and horizontal diameter increased, while longitudinal diameter remained relatively stable. From a nutritional and flavor perspective, the contents of soluble solids and sugars (sucrose, fructose, glucose) significantly increased as the fruit transitioned from green to blue maturity stages, notably enhancing sweetness (Wu et al. 2024; Villagra et al. 2024). In contrast, the titratable acid content declined (Li et al. 2020), and Vitamin C consistently increased (Farina et al. 2020). VOC analyses are critical for objectively characterizing aroma shifts, and future work may further integrate electronic tongue measurements to strengthen the linkage between human perception and instrumental profiles (Huang et al. 2024; Wei et al. 2025).

### 4.2 Dynamics of VOCs and Underlying Metabolic Shifts

Blueberries contain numerous VOCs, but only those surpassing their flavor thresholds significantly impact aroma (Farneti et al. 2017). Aldehydes such as hexanal, nonanal, and octanal play a significant role in the “Legacy” blueberry aroma by contributing floral notes reminiscent of citrus, rose, and jasmine (Sun et al. 2024; Wang et al. 2024). The constant presence of (*E*)‐2‐hexenal with OAV > 1 across all stages confirms its status as a foundational aroma component and primary maturity indicator for this cultivar (Toledo Guerrero et al. 2024). The transition from “green” to “ripe” notes reflects distinct metabolic pathways. In early maturity stages (Maturity Stage I), the predominance of “green” compounds like humulene and (*E*)‐3‐hexenoic acid correlates with lipoxygenase pathway activity (following the enzymatic oxidation of linoleic and linolenic acids), defining sharp, unripe characteristics (Liang et al. 2020). The significant negative correlation of these aldehydes with SSC and their positive correlation with acidity further support this. As ripening progresses, the emergence of 1‐octen‐3‐one adds complexity with earthy and vegetable notes (Leffingwell and Leffingwell 1991; Wang et al. 2008). Furthermore, the consistent presence of β‐myrcene enhances aroma depth, contributing sweet and creamy undertones known to increase the complexity of various berry flavor profiles (Van Gemert 2011; Sun and Chen 2016).

As the fruit matures beyond the early stages, a coordinated softening and aroma maturation occurs, likely driven by common upstream regulators in cell wall degradation and VOC biosynthesis (Xi et al. 2023). The action of alcohol dehydrogenase probably converts C6 aldehydes into corresponding alcohols, reducing pungency and enhancing fruity characteristics (Chang et al. 2013). Concurrently, the upregulation of alcohol acyltransferase facilitates ester formation, whereas terpene synthase activity promotes the production of compounds like linalool (Shalit et al. 2001). At Maturity Stage IV, these metabolic shifts result in a synergistic interplay of sweetness, acidity, texture, and aroma development. During this stage, the positive correlation between hexanal and SSC suggests that sweetness development is complemented by concurrent floral aroma enhancement. Various factors, including genetic variations and orchard management, can further influence these pathways (Cristea et al. 2024; Xu et al. 2025; Dominici et al. 2025), necessitating cultivar‐specific evaluations.

### 4.3 Potential Biochemical Markers for Maturity Assessment

Our analysis showed that ripe blueberries possessed high levels of hexanal and (*E*)‐2‐hexenal, followed by (*E*)‐2‐hexen‐1‐ol, linalool, and L‐α‐terpineol. Integrating clustering analysis with OPLS‐DA allowed us to identify these key differential aroma compounds across maturity stages. Beyond traditional visual cues, these specific VOCs can serve as potential biochemical markers for non‐destructive maturity assessment. In a similar approach, Shi et al. (2025) identified hexyl acetate as an important marker for the ripening of “Yuluxiang” pears, suggesting that fruit maturity could be determined via its rapid detection. Based on our OPLS‐DA results, for the “Legacy” blueberry, a high ratio of hexanal to (*E*)‐2‐hexenal could act as a quantitative indicator of optimal maturity stage. The development of rapid detection technologies, such as portable electronic noses or specific gas sensors calibrated to these key VOC markers, could revolutionize harvest timing by providing an objective, real‐time measure of the flavor peak, surpassing the subjectivity of color assessment alone. Such technology has already shown promise in fruit varieties like pitaya and grapes (Aleixandre et al. 2015; da Silva Ferreira et al. 2023).

### 4.4 Implications for Processing and Future Perspectives

From a processing and utilization perspective, the present VOC/OAV‐based characterization provides a practical basis for raw‐material grading. Previous studies have shown that blueberry fermentation (e.g., wine and fermented juice) markedly reshapes the volatile profile. Shifts in esters, terpenes, and selected alcohols are key drivers of fruity‐floral versus green/fermented aroma notes; selecting maturity stages enriched in fruity‐floral contributors and with reduced “green‐note” aldehydes is likely to improve the flavor quality and consumer acceptance of fermented products (Liu et al. 2025). Furthermore, yeast/lactic‐acid‐bacteria fermentations have been reported to increase ester‐ and terpene‐related aromas while decreasing grassy or pungent aldehydes, leading to a more harmonious aromatic profile (Wang, Wei, et al. 2023). Thus, the key discriminant VOC markers identified here (e.g., (*E*)‐2‐hexenal, hexanal, linalool) may serve not only for harvest maturity grading of fresh fruit but also as rapid indicators for selecting suitable raw materials for juice, wine, and other processed products (Gu et al. 2022). Future research combining metabolomics and transcriptomics could further elucidate the molecular mechanisms governing these aroma changes. Additionally, comparing multiple cultivars would help clarify the genetic and environmental influences on stage‐specific VOC signatures.

## 5 Conclusions

This study comprehensively characterized the physicochemical and volatile organic compound (VOC) profiles of “Legacy” blueberries across five maturity stages. As maturation progressed, nutritional quality improved—marked by increased soluble solids and vitamin C—while firmness and acidity decreased. Although early stages exhibited higher VOC diversity, optimal aromatic complexity emerged during mid‐to‐late ripening, driven synergistically by key compounds such as linalool, (*E*)‐2‐hexenal, nonanal, and β‐myrcene. Crucially, this research establishes specific VOCs not merely as flavor contributors, but as reliable biochemical markers for ripeness. Integrating these profiles indicates that Maturity Stage IV is the optimal harvest window, achieving an ideal balance of structural integrity, peak nutrition, and maximum aroma. Ultimately, these stage‐specific VOC signatures provide a practical basis for the blueberry industry to optimize harvest timing and raw material selection for both fresh consumption and processing. Future multi‐omics research will further elucidate the molecular networks governing these critical flavor transitions.

## Author Contributions

**Wenkuan Zhang:** investigation, visualization, writing – original draft, formal analysis. **Chaoshuang Jia:** investigation, conceptualization, data curation. **Qiang Yue:** resources, methodology. **Yang Wang:** writing – review and editing, data curation, methodology. **Wenhui Wang:** writing – review and editing, funding acquisition, investigation, validation. **Zhihua Wang:** methodology, writing – review and editing, funding acquisition, project administration, resources, supervision. **Shumin Zhang:** investigation, software, validation. **Yanmin Du:** project administration, supervision.

## Funding

This project was supported by the key Research and Development Program of the Ministry of Science and Technology of China during the 14th Five Year Plan Period (2022YFD1600504‐3); China Academy of Agricultural Sciences Science and Technology Innovation Project (CAAS‐ASTIP‐RIP).

## Conflicts of Interest

The authors declare no conflicts of interest.

## Supporting information


**Table S1:** Reference standards for the evaluation of blueberry flavor attributes by trained panels.
**Table S2:** Sensory characteristics of blueberry at different maturity stages.

## Data Availability

The authors have nothing to report.
