# Supplementary material for: Influence of Maturity Stage at Harvest on the Fruit Quality and Volatile Organic Compounds of “Legacy” Blueberry
Source: Food Sci Nutr. 2026 Apr 16;14(4):e71792. doi: 10.1002/fsn3.71792 (PMC13084260; doi:10.1002/fsn3.71792)
Supplement: Supplementary file 1 — Table S1: Reference standards for the evaluation of blueberry flavor attributes by trained panels. Table S2: Sensory characteristics of blueberry at different maturity stages. [file FSN3-14-e71792-s001.docx]

**Supplementary Material**

**Influence of maturity stage at harvest on the fruit quality and volatile organic compounds of 'Legacy' blueberry**

**Wenkuan Zhang ^1,2,3^, Zhihua Wang ^1,2,3^,Wenhui Wang ^1,2,3^,Chaoshuang Jia ^1,2,3^,**

**Yang Wang ^1,2,3^,Shumin Zhang ^1,2,3^,Qiang Yue^1,2,3^,Yanmin Du^1,2,3^**

1 Institute of Pomology, Chinese Academy of Agricultural Sciences, Xingcheng 125100, China

2 Key Laboratory of Germplasm Resources Utilization of Horticultural Crops, Ministry of Agriculture and Rural Affairs, Xingcheng 125100, China

3 Key Laboratory of Fruits Storage and Processing of Liaoning Province, Xingcheng 125100, Chi

**Table S1. Reference standards for the evaluation of blueberry flavor attributes by trained panels.**

| **Attribute** | **Definition** | **Reference Standard** |
| --- | --- | --- |
| ****Overall Acceptance**** | Overall degree of liking. | Based on a weighted average impression of all other attributes. |
| ****Crispness**** | Force and sound upon biting | Fresh apple (e.g., Fuji apple). |
| ****Juiciness**** | Amount of liquid released | Watermelon (95% water content). |
| ****Sourness**** | Taste associated with acidity | 0.07% Malic acid solution. |
| ****Sweetness**** | Taste associated with sugar | 3% Sucrose solution. |
| ****Astringency**** | Drying, puckering sensation | Light black tea  (0.04% Tannic acid solution). |
| ****Green**** | Aroma of fresh vegetation | Hexanal. |
| ****Fruity**** | Aroma of ripe fruit | Hexyl acetate. |
| ****Floral**** | Aroma of flowers | Linalool. |
| ****Fragrant**** | Pleasant, diffuse aroma | Ripe banana. |

**Table S2. Sensory characteristics of blueberry at different maturity stages**

| **Attribute** | **Maturity I** | **Maturity II** | **Maturity III** | **Maturity IV** | **Maturity V** |
| --- | --- | --- | --- | --- | --- |
| **Overall Acceptance** | 2.5^a^ | 4.5^b^ | 6.5^c^ | **8.3^d^** | 7.8^cd^ |
| **Crispness** | 8.5^a^ | 7.5^b^ | 6.5^c^ | **5.2^d^** | 3.5^e^ |
| **Juiciness** | 3.0^a^ | 4.2^b^ | 5.0^c^ | **4.8^bc^** | 4.5^b^ |
| **Sourness** | 8.5^a^ | 7.0^b^ | 4.5^c^ | **2.1^d^** | 1.5^d^ |
| **Sweetness** | 1.5^a^ | 3.5^b^ | 6.0^c^ | **7.8^d^** | 8.5^d^ |
| **Astringency** | 8.0^a^ | 6.5^b^ | 3.5^c^ | **1.8^d^** | 1.5^d^ |
| **Green** | 8.8^a^ | 7.2^b^ | 5.5^c^ | **4.2^d^** | 2.5^e^ |
| **Fruity** | 1.8^a^ | 3.8^b^ | 5.8^c^ | **6.8^d^** | 7.0^d^ |
| **Floral** | 2.0^a^ | 4.0^b^ | 5.0^c^ | **4.5^bc^** | 3.8^b^ |
| **Fragrant** | 2.2^a^ | 3.5^b^ | 4.2^c^ | **3.6^bc^** | 3.0^b^ |

Note:The different lowercase letters (a-e) indicate significant differences among treatment groups (*P* < 0.05).
